# Vitamin E Modulates Hepatic Extracellular Adenosine Signaling to Attenuate Metabolic Dysfunction-Associated Steatotic Liver Disease (MASLD)

**DOI:** 10.3390/ijms27020614

**Published:** 2026-01-07

**Authors:** Mengting Shan, Magdeline E. Carrasco Apolinario, Tomoko Tokumaru, Kenshiro Shikano, Phurpa Phurpa, Ami Kato, Hitoshi Teranishi, Shinichiro Kume, Nobuyuki Shimizu, Tatsuki Kurokawa, Takatoshi Hikida, Toshikatsu Hanada, Yulong Li, Reiko Hanada

**Affiliations:** 1 Department of Physiology, Faculty of Medicine, Oita University, Yufu 879-5593, Oita, Japan; m21d9025@oita-u.ac.jp (M.S.); mecarrasco@oita-u.ac.jp (M.E.C.A.); kshikano@oita-u.ac.jp (K.S.); m22d9018@oita-u.ac.jp (P.P.); m2041025@oita-u.ac.jp (A.K.); teranishi@oita-u.ac.jp (H.T.); s-kume@oita-u.ac.jp (S.K.); tkurokawa@oita-u.ac.jp (T.K.); 2 Division of Gastroenterology, Department of Internal Medicine, Faculty of Medicine, Oita University, Yufu 879-5593, Oita, Japan; tomo-tokumaru@oita-u.ac.jp; 3 Department of Biochemistry and Molecular Genetics, Faculty of Medicine, Oita University, Yufu 879-5593, Oita, Japan; ns5725@oita-u.ac.jp (N.S.); thanada@oita-u.ac.jp (T.H.); 4 Laboratory for Advanced Brain Functions, Institute for Protein Research, Osaka University, Suita 565-0871, Osaka, Japan; hikida@protein.osaka-u.ac.jp; 5 State Key Laboratory of Membrane Biology, School of Life Sciences, Peking University, Beijing 100871, China; yulongli@pku.edu.cn

**Keywords:** metabolic dysfunction-associated steatotic liver disease (MASLD), vitamin E, extracellular adenosine (eAdo), GRAB_Ado_ sensor, zebrafish model

## Abstract

Metabolic dysfunction-associated steatotic liver disease (MASLD) involves early disturbances such as excessive lipid accumulation, sterile inflammation, and hepatocellular stress. The results of recent studies have highlighted extracellular ATP and its metabolite adenosine (Ado) as damage-associated molecular patterns (DAMPs) that drive inflammation, endoplasmic reticulum (ER) stress, and steatosis, contributing to MASLD progression. Although vitamin E is clinically used for its antioxidant and anti-inflammatory properties, it remains unclear whether its therapeutic effects involve modulation of DAMP-associated signaling. To address this gap, we used transgenic zebrafish expressing a liver-specific G-protein-coupled receptor activation-based adenosine sensor (GRAB_Ado_). We found that a high-cholesterol diet markedly increased hepatic extracellular Ado levels, combined with inflammatory and ER stress-associated gene expression. Vitamin E significantly reduced extracellular Ado levels and hepatic lipid accumulation. Based on RNA sequencing results, vitamin E restored the expression of genes encoding calcium-handling proteins, including *atp2a1* and *atp1b1b*. These genes encode components of the sarco/ER Ca^2+^-ATPase (SERCA) machinery, which is essential for maintaining ER Ca^2+^ homeostasis and preventing stress-induced hepatic injury. CDN1163-mediated SERCA activation phenocopied the protective effect of vitamin E, supporting a Ca^2+^-dependent mechanism. Together, these findings highlight extracellular Ado signaling and impaired SERCA-mediated Ca^2+^ regulation as early drivers of MASLD and demonstrate that vitamin E ameliorates steatosis by targeting both pathways.

## 1. Introduction

Metabolic dysfunction-associated steatotic liver disease (MASLD) is one of the most prevalent metabolic disorders worldwide and imposes a substantial socioeconomic burden [1,2]. MASLD corresponds to the metabolic steatotic stage and is the basis for the development of metabolic dysfunction-associated steatohepatitis (MASH), which can subsequently progress to fibrosis, cirrhosis, and hepatocellular carcinoma [3]. Because MASLD, defined by hepatic steatosis, constitutes the earliest and fully reversible phase of this disease spectrum, timely intervention is essential to prevent its progression. When the condition advances to MASH, only partial reversibility is possible, and persistent injury can drive the further transition to fibrosis, cirrhosis, and ultimately hepatocellular carcinoma, stages that are largely irreversible. Therefore, clarifying the molecular mechanisms underlying MASLD initiation, particularly those operating at the steatotic stage, is vital for preventing subsequent, more severe hepatic complications. Although the pathogenesis of MASLD has been widely investigated, the contribution of sterile inflammation to early steatosis remains insufficiently understood.

Growing evidence indicates that sterile inflammation plays a central role in the initiation of MASLD. Damage-associated molecular patterns (DAMPs), released from metabolically stressed or dying hepatocytes, including extracellular ATP (eATP) and its metabolite, extracellular adenosine (eAdo), activate purinergic receptors and amplify inflammatory and metabolic stress responses in both autocrine and paracrine manners [4,5,6]. These extracellular nucleotides are increasingly recognized as critical molecular links between metabolic dysregulation and hepatic inflammation. Nevertheless, despite this recognition, the specific contribution of the hepatic eATP/eAdo axis during the earliest phase of steatosis—and whether this pathway may serve as a viable therapeutic target—remains incompletely defined.

Vitamin E, a potent lipophilic antioxidant, has drawn considerable attention for its ability to suppress oxidative stress and inflammation [7,8]. The results of multiple clinical and experimental studies have demonstrated that it can improve hepatic steatosis and ameliorate liver injury in MASLD [9,10,11,12,13]. Nevertheless, the authors of these studies primarily examined advanced disease stages, and the mechanisms by which vitamin E exerts its hepatoprotective effects, particularly whether it modulates DAMP-associated eAdo signaling, remain unclear.

To address this gap, we used transgenic zebrafish expressing a liver-specific G-protein-coupled receptor activation-based adenosine (GRAB_Ado_) sensor [4,14] to monitor hepatic eAdo dynamics in vivo during high-cholesterol diet (HCD)-induced steatosis. We observed that vitamin E reduced hepatic eAdo accumulation and alleviated steatosis. To further elucidate the mechanisms underlying this effect, we performed RNA sequencing using the livers of vitamin E-treated zebrafish. This analysis revealed that vitamin E could restore the expression of genes encoding intracellular Ca^2+^-handling proteins, including *atp2a1* and *atp1b1b*, which encode components of the sarco/endoplasmic reticulum (ER) Ca^2+^-ATPase (SERCA) machinery, essential for maintaining the ER Ca^2+^ balance and preventing stress-induced hepatocellular dysfunction. The pharmacological enhancement of SERCA activity using CDN1163 phenocopied the protective effects of vitamin E, suggesting that the normalization of Ca^2+^ homeostasis functions downstream of, or in parallel with, eAdo regulation. Together, these findings highlight a previously unrecognized mechanistic association between eAdo signaling and intracellular Ca^2+^ regulation in the context of vitamin E-mediated hepatoprotection, offering new insight into therapeutic strategies aimed at the earliest molecular events driving MASLD.

## 2. Results

### 2.1. Establishment of an HCD-Induced MASLD Model Using Zebrafish and Evaluation of Hepatic Extracellular Adenosine Dynamics

**Figure 1:**
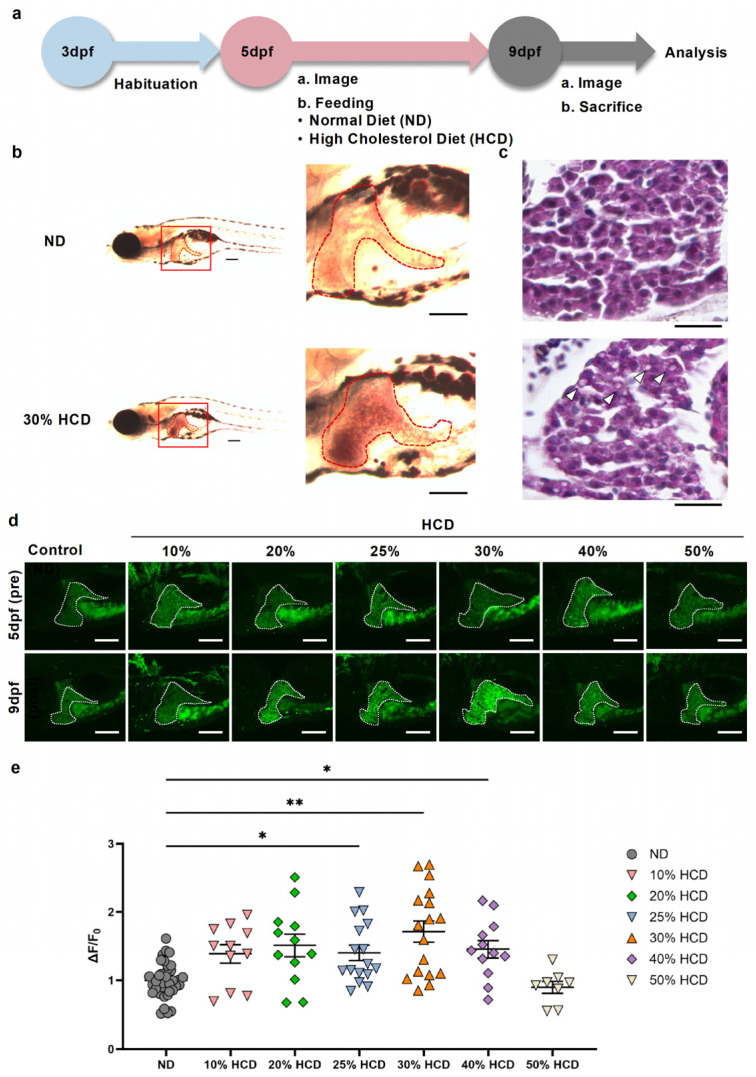
**A high-cholesterol diet (HCD) promotes hepatic steatosis and increases extracellular adenosine (eAdo) levels**. (**a**) Experimental protocol. Zebrafish larvae were habituated until 3 days post fertilization (dpf), followed by feeding with either a normal diet (ND) or a high-cholesterol diet (HCD) from 5 to 9 dpf. At 9 dpf, larvae were subjected to imaging and tissue analysis. (**b**) Representative images of Oil Red O staining of liver tissue (outlined in red) in wild-type larvae at 9 dpf, maintained on an ND or a 30% HCD. Sagittal sections are shown at lower and higher magnification. Scale bar = 200 µm. (**c**) Hematoxylin and eosin (H&E)-stained liver sections of ND and 30% HCD group larvae. Hepatic lipid droplets are observed in HCD-fed larvae (white arrowheads). Scale bar = 40 μm. (**d**) Representative images of liver GFP fluorescence in G-protein-coupled receptor activation-based adenosine sensor (GRAB_Ado_)-expressing zebrafish larvae at 9 dpf fed an ND or varying concentrations of an HCD (10–50%). The liver area is outlined by a white dashed line. Scale bar = 100 μm. (**e**) Quantification of the change in the GFP fluorescence intensity in the livers of GRAB_Ado_-expressing larvae fed an ND or HCD (10–50%). Data represent the ΔF/F_0_ from 8 to 38 larvae per group (ND, *n* = 38; 10% HCD, *n* = 11; 20% HCD, *n* = 12; 25% HCD, *n* = 16; 30% HCD, *n* = 17; 40% HCD, *n* = 12; 50% HCD, *n* = 8). Data are shown as the mean  ±  SEM. * *p * <  0.05; ** *p*  <  0.01 vs. ND.

To investigate the relationship between eAdo dynamics and the development of MASLD, zebrafish larvae were fed either a normal diet (ND) or an HCD based on the timeline shown in Figure 1a. A morphological evaluation using Oil Red O and H&E staining revealed that larvae fed a 30% HCD had enlarged livers with markedly increased lipid accumulation compared with those of ND-treated controls (Figure 1b,c). These findings confirmed that a 30% HCD could robustly induce hepatic steatosis, recapitulating early MASLD-like phenotypes, in zebrafish. To assess hepatic eAdo dynamics with varying dietary cholesterol concentrations, we utilized transgenic zebrafish expressing the liver-specific GRAB_Ado_ sensor (Figure 1d). Larvae were exposed to diets containing 10%, 20%, 25%, 30%, 40%, or 50% cholesterol. The GRAB_Ado_ fluorescence intensity was significantly elevated in larvae fed a 25–40% HCD, particularly in the 30% and 40% HCD groups, compared with the ND control groups (Figure 1d,e). Moreover, the hepatic GFP intensity showed a concentration-dependent increase in response to higher cholesterol contents (Figure 1e). Based on both the morphological and GRAB_Ado_ fluorescence responses, a 30% HCD was selected for subsequent experiments, as it consistently induced significant hepatic steatosis, while larval viability and reproducibility were maintained.

### 2.2. HCD Induces Inflammatory, Inflammasome-Related, and ER-Stress Gene Expression in the Zebrafish Liver

**Figure 2:**
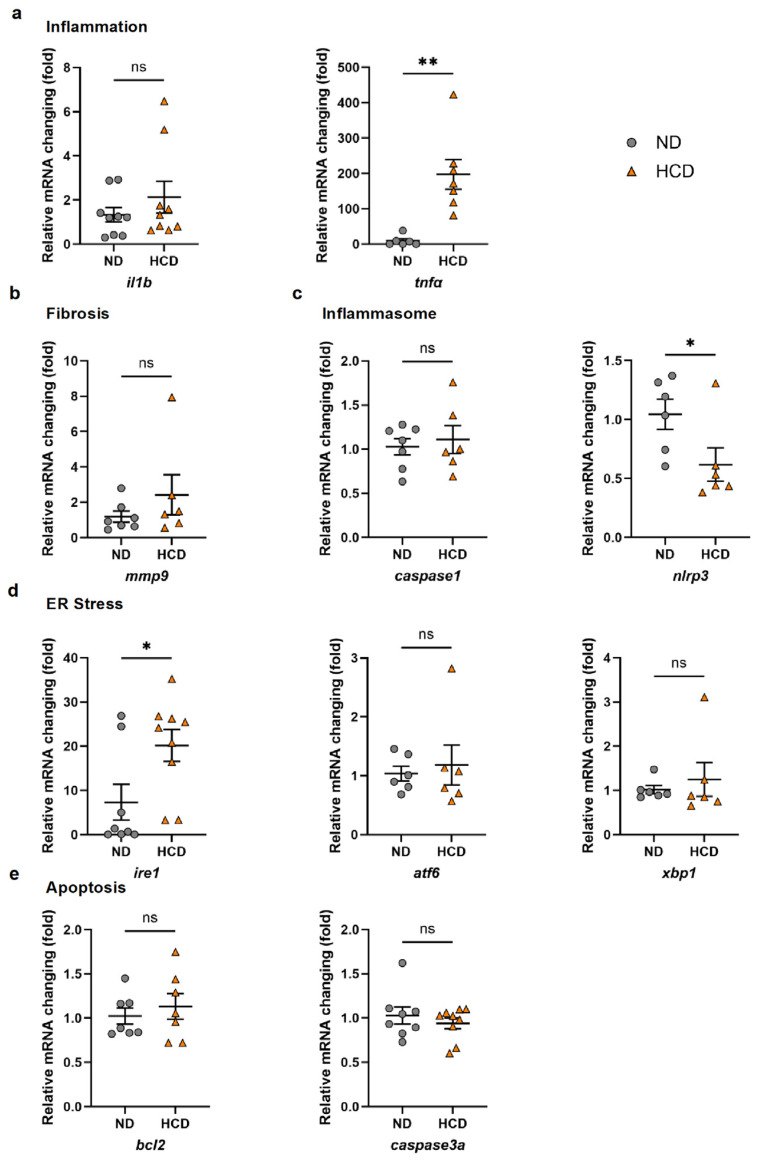
**A high-cholesterol diet (HCD) selectively modulates inflammatory, inflammasome-related, and stress-response gene expression in zebrafish larvae**. (**a**) Relative mRNA expression of proinflammatory cytokine-encoding genes (*il1b* and *tnfa*) in zebrafish larvae fed a normal diet (ND) or a 30% HCD (ND, *n* = 6–9; 30% HCD, *n* = 7–9). HCD feeding significantly increased *tnfa* expression; in comparison, *il1b* remained unchanged. (**b**) Fibrosis-related gene expression (*mmp9*) in the ND and HCD groups (ND, *n* = 7; 30% HCD, *n* = 6). No significant difference was observed. (**c**) Expression of inflammasome-associated genes (*caspase1* and *nlrp3*) in the ND and HCD groups (ND, *n* = 6–7; 30% HCD, *n* = 6). Whereas *caspase1* expression showed no significant change, *nlrp3* expression was significantly downregulated in HCD-exposed larvae. (**d**) Expression of endoplasmic reticulum (ER) stress markers (*atf6* and *xbp1*) in the ND and HCD groups (ND, *n* = 6; 30% HCD, *n* = 6). Levels of both genes showed slight, non-significant increases following HCD treatment. (**e**) Apoptosis-related gene expression (*bcl2* and *caspase3a*) exhibited no significant differences between ND- and HCD-fed larvae (ND, *n* = 7–8; 30% HCD, *n* = 7–9). Each dot represents 20–25 zebrafish larvae. Data represent the mean ± SEM. * *p*  <  0.05; ** *p*  <  0.01 vs. ND; n.s., non-significant.

To further characterize the molecular alterations associated with HCD-induced steatosis, we quantified hepatic mRNA levels of inflammation-, fibrosis-, inflammasome-, ER stress-, and apoptosis-related genes in zebrafish larvae. The expression of *tnfα*, encoding a proinflammatory cytokine, was significantly increased in HCD-treated larvae compared with that in the ND group (Figure 2a). Among fibrosis-related genes, *mmp9* showed an increasing trend after HCD administration, though no significant difference was observed. (Figure 2b). We next examined genes encoding components of the inflammasome pathway. Whereas *caspase1* expression showed a slight upward trend, *nlrp3* expression was unexpectedly reduced in HCD-treated larvae relative to the controls (Figure 2c), suggesting the complex or stage-dependent regulation of inflammasome signaling under steatotic conditions. An analysis of ER-stress markers revealed a significant upregulation of the expression of *ire1*, whereas *atf6* and *xbp1* mRNA levels showed minor increases, without reaching statistical significance (Figure 2d). These findings indicate that HCD feeding induces selective activation of the IRE1-mediated unfolded protein response in the zebrafish liver. In contrast, the expression of apoptosis-related genes (*bcl2a* and *caspase3a*) did not exhibit pronounced changes between groups (Figure 2e). Collectively, these results demonstrate that a 30% HCD triggers hepatic steatosis accompanied by enhanced inflammatory and ER-stress responses, establishing a MASLD-like molecular phenotype in zebrafish larvae.

### 2.3. Vitamin E Alleviates Hepatic Steatosis by Reducing eAdo Levels

**Figure 3:**
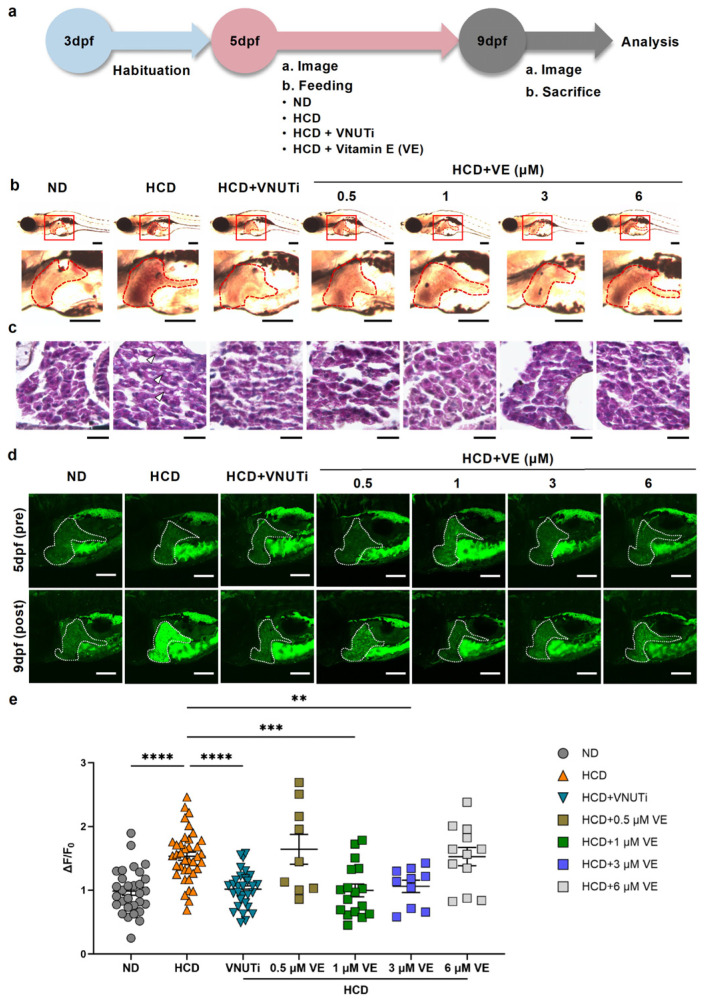
**Vitamin E attenuates high-cholesterol diet (HCD)-induced hepatic steatosis and reduces extracellular adenosine (eAdo) levels in zebrafish larvae**. (**a**) Schematic overview of the treatment protocol. Zebrafish larvae were habituated until 3 days post fertilization (dpf) and subsequently fed with a normal diet (ND), HCD, HCD + VNUT inhibitor (VNUTi), or HCD supplemented with vitamin E (0.5–6 µM) from 5 to 9 dpf. Larvae were collected for imaging or tissue analysis at 9 dpf. (**b**) Representative images of Oil Red O staining of liver tissues (outlined in red) in ND-, HCD-, HCD + VNUTi-, and HCD + vitamin E-treated larvae at 9 dpf. Sagittal sections are shown at lower and higher magnification. Scale bar = 200 µm. Red boxed regions indicate the liver area, which appears to be enlarged and lipid-laden in HCD-fed larvae compared with the phenotype of ND controls. (**c**) Hematoxylin and eosin (H&E)-stained liver sections from ND, HCD, HCD + VNUTi, and HCD + vitamin E groups. Lipid droplets are evident in HCD-fed larvae (white arrowheads), whereas VNUT inhibition and vitamin E treatment reduced intracellular lipid accumulation. Scale bar = 40 µm. (**d**) Representative GFP fluorescence images of GRAB_Ado_-expressing zebrafish larvae. The liver-specific eAdo level (ΔF/F_0_) was markedly increased by HCD feeding and was attenuated by VNUTi and vitamin E in a dose-dependent manner. The liver region is outlined with a white dotted line. Scale bar = 100 µm. (**e**) Quantification of liver GFP fluorescence intensity (ΔF/F_0_) in GRAB_Ado_-expressing larvae across all treatment groups. Data represent the ΔF/F_0_ from 9 to 38 larvae per group (ND, *n* = 30; HCD, *n* = 38; HCD + VNUTi, *n* = 30; HCD + 0.5µM VE, *n* = 9; HCD + 1µM VE, *n* = 17; HCD + 3µM VE, *n* = 10; HCD + 6µM VE, *n* = 12). Data are presented as the mean  ±  SEM. ** *p*  <  0.01; *** *p*  <  0.001; **** *p*  <  0.0001 vs. ND; n.s., non-significant.

Excessive cholesterol accumulation in hepatocytes promotes ER stress, cell damage, and ferroptosis [15], all of which contribute to the progression of MASLD/MASH. Although vitamin E is widely recognized for its antioxidant and anti-inflammatory properties [16], its impact on eAdo dynamics in vivo has not been clearly demonstrated. To investigate whether vitamin E modulates hepatic eAdo signaling during steatosis, we treated GRAB_Ado_-expressiong zebrafish larvae exhibiting HCD-induced liver steatosis with vitamin E (1–10 μM). In parallel, we administered a vesicular nucleotide transporter inhibitor (VNUTi), which reduces vesicular ATP release and thereby decreases eAdo production, as a positive control for both eAdo reduction and steatosis improvement (Figure 3a) [4]. Oil Red O and H&E staining revealed that both VNUTi and vitamin E (1 and 3 μM) markedly attenuated hepatic lipid accumulation in HCD-fed larvae (Figure 3b,c). However, higher concentrations of vitamin E did not further improve steatosis. We next evaluated hepatic eAdo dynamics in GRAB_Ado_-expressing zebrafish. Consistent with their morphological effects, VNUTi and vitamin E, at 1 and 3 μM, significantly reduced liver GFP fluorescence compared with that in untreated HCD-fed controls (Figure 3d,e). In contrast, other vitamin E concentrations did not alter the GRAB_Ado_ fluorescence intensity. Because eAdo levels reflect extracellular ATP release—with elevated ATP/Ado promoting sterile inflammation in MASLD [5]—the observed reduction in GRAB_Ado_ fluorescence indicates that vitamin E suppresses pathological eAdo signaling during steatosis. Together, these results demonstrate that vitamin E ameliorates early-stage MASLD-like hepatic steatosis in vivo, at least in part through a reduction in eAdo levels.

### 2.4. Vitamin E Modulates Intracellular Calcium-Related Gene Expression in HCD-Induced MASLD

**Figure 4:**
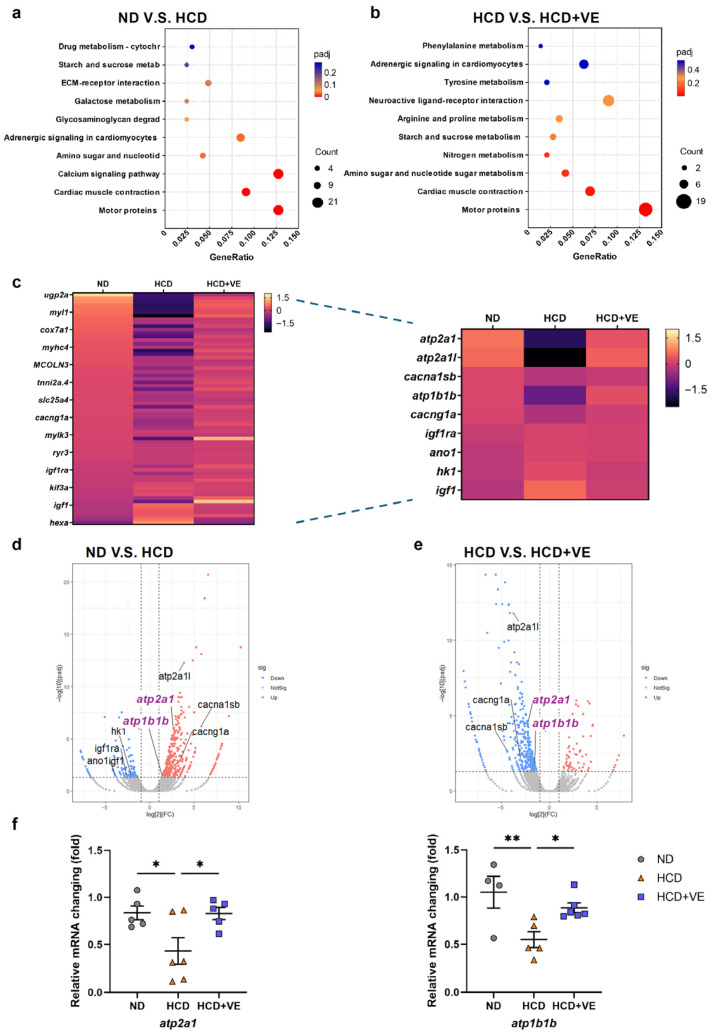
**Transcriptomic profiling reveals high-cholesterol diet (HCD)-induced alterations in hepatic gene expression and their partial normalization after vitamin E treatment**. (**a**) Dot-plot of enriched KEGG pathways comparing normal diet (ND) and 30% HCD groups, showing the enrichment of pathways associated with inflammation, metabolic dysregulation, calcium signaling, and endoplasmic reticulum (ER) stress in HCD-fed larvae. (**b**) Dot-plot of enriched KEGG pathways comparing HCD and HCD + vitamin E (VE) groups. Vitamin E treatment attenuated multiple HCD-enriched pathways, including inflammatory and metabolic signaling cascades. (**c**) Heatmap of differentially expressed genes (DEGs) among ND, HCD, and HCD + VE groups. The expression of a subset of genes altered by the HCD (*atp2a1*, *atp1b1b*, *atp2a1l*, *igf1*, and *igf1ra*) showed the partial or complete restoration toward ND levels following VE treatment. (**d**) Volcano plot comparing ND versus HCD groups. Genes for which expression was significantly upregulated or downregulated by HCD are highlighted, including those encoding calcium pump regulators, such as *atp2a1*. (**e**) Volcano plot comparing HCD versus HCD + VE groups. VE supplementation reversed the expression trends of specific HCD-responsive genes, including *atp1b1b*. (**f**) qRT-PCR-based validation of selected DEGs (*atp2a1* and *atp1b1b*) across the ND, HCD, and HCD + VE groups, confirming the transcriptomic trends identified via RNA-seq (ND, *n* = 4–5; HCD, *n* = 5–6; HCD + VE, *n* = 5–6). Each dot represents 102–180 livers of zebrafish larvae. Data represent the mean ± SEM. * *p* < 0.05; ** *p* < 0.01 vs. ND or HCD.

To further elucidate the molecular mechanisms through which vitamin E alleviates the MASLD-like pathology, we performed RNA sequencing using livers collected from zebrafish larvae, 9 days post fertilization (dpf), fed an ND, HCD, or HCD supplemented with vitamin E (3 μM). Among these three groups, 9134 differentially expressed genes (DEGs) were identified. To determine the biological significance of these DEGs, we performed KEGG pathway enrichment analysis. Pathways associated with cardiac muscle contraction and motor protein function were among the top 10 significantly enriched pathways (Figure 4a,b). Although classical “calcium signaling pathways” were not included in the top 10 terms, the expression of several genes within these enriched pathways—including *atp2a1* and *atp1b1b*, which encode components of SERCA-mediated Ca^2+^ handling—showed marked alterations, suggesting that the intracellular Ca^2+^ regulatory machinery is affected downstream of vitamin E treatment. Hierarchical clustering via heatmap visualization further demonstrated distinct expression patterns among the groups (Figure 4c). Moreover, a volcano plot analysis revealed that, relative to levels in the HCD group, the ND group contained 411 upregulated and 113 downregulated genes, whereas vitamin E treatment resulted in upregulation of 405 and downregulation of 71 genes compared to the levels of HCD-fed larvae (Figure 4d,e). From these datasets, we identified nine genes associated with glucose metabolism, carbohydrate metabolism, or intracellular Ca^2+^ homeostasis (*atp1b1b*, *cacna1sb*, *cacng1a*, *atp2a1/serca*, *atp2a1l*, *ano1*, *igf1ra*, *hk1*, and *igf1*) that were consistently modulated by vitamin E (Figure 4c).

Among these genes, *atp2a1* (SERCA1) and *atp1b1b*, both encoding key regulators of intracellular Ca^2+^ transport, were selected for validation via qPCR. HCD feeding significantly reduced the expression of both genes; in comparison, vitamin E treatment restored their expression to levels comparable to or higher than those in ND controls (Figure 4f). These findings indicate that vitamin E counteracts the HCD-induced MASLD-like pathology, at least in part, by modulating genes that are essential for intracellular Ca^2+^ homeostasis, implicating SERCA-related pathways as downstream targets of vitamin E.

### 2.5. SERCA Activation Ameliorates MASLD-like Steatosis by Reducing Hepatic eAdo Levels

**Figure 5:**
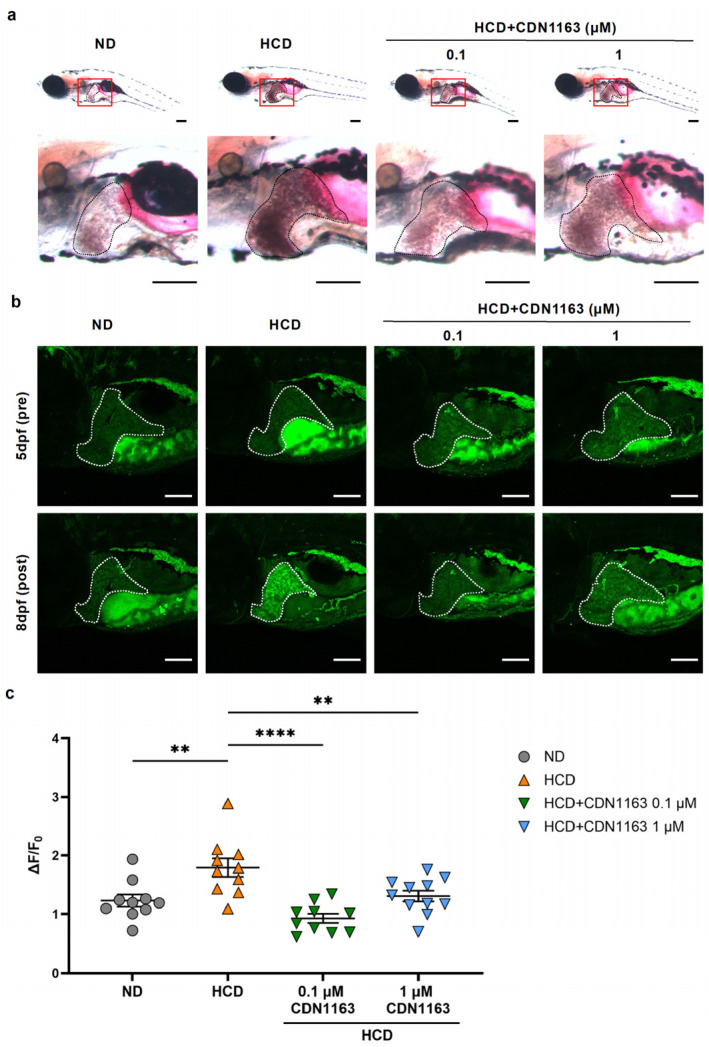
**Activation of SERCA by CDN1163 ameliorates high-cholesterol diet (HCD)-induced hepatic steatosis and reduces extracellular adenosine (eAdo) levels in zebrafish larvae**. (**a**) Representative images of Oil Red O staining of liver tissue (outlined in black) in groups administered a normal diet (ND), HCD, or HCD supplemented with the SERCA activator CDN1163 (0.1 or 1 µM) from 5 to 8 days post fertilization (dpf). Red boxed regions indicate the liver area. HCD-fed larvae exhibited pronounced hepatic enlargement and lipid accumulation, which were suppressed by CDN1163 treatment in a dose-dependent manner. Sagittal sections are shown at lower and higher magnification. Scale bar = 200 µm. (**b**) Representative GFP fluorescence images of GRAB_Ado_-expressing zebrafish larvae showing eAdo levels in the liver. HCD feeding markedly increased the ΔF/F_0_ intensity, indicating increased eAdo levels; in comparison, CDN1163 administration attenuated this elevation. Liver regions are outlined with a white dotted line. Scale bar = 100 µm. (**c**) Quantification of liver GFP fluorescence intensity (ΔF/F_0_) across ND, HCD, and CDN1163-treated groups. CDN1163 significantly suppressed the HCD-induced increase in the ΔF/F_0_. Data represent the ΔF/F_0_ from 10 to 11 larvae per group (ND, *n* = 10; HCD, *n* = 10; HCD + 0.1µM CDN1163, *n* = 10; HCD + 1µM CDN1163, *n* = 11). Data are presented as the mean ± SEM. ** *p* < 0.01; **** *p* < 0.0001 vs. ND or HCD.

SERCA is the principal pump responsible for transporting Ca^2+^ from the cytosol to into the ER, thereby maintaining intracellular Ca^2+^ homeostasis. Our RNA-seq analysis results indicated that *atp2a1* (SERCA) expression was significantly downregulated in zebrafish with an HCD-induced MASLD-like pathology, consistent with previous reports demonstrating that impaired SERCA activity contributes to steatosis and ER stress in metabolic liver disease models [17]. Although pharmacological SERCA activation has been shown to restore the ER Ca^2+^ balance and attenuate hepatic lipid accumulation in mammalian models, whether SERCA influences eAdo dynamics in vivo remains unknown. To assess this factor, we administered the SERCA allosteric activator CDN1163 to wild-type or GRAB_Ado_-expressiong zebrafish larvae exhibiting HCD-induced hepatic steatosis. CDN1163 treatment markedly reduced hepatic lipid deposition, as demonstrated by a decrease in Oil Red O-positive lipid accumulation compared to that in the untreated MASLD-like larvae (Figure 5a). We next evaluated hepatic eAdo levels based on GRAB_Ado_ fluorescence. CDN1163 significantly decreased the GFP fluorescence intensity in the liver, with the strongest effect observed with 0.1 μM (Figure 5b,c). This reduction in eAdo occurred concomitantly with the improvement in steatosis. Collectively, these findings demonstrate that SERCA activation is sufficient to attenuate the MASLD-like pathology in vivo, at least in part by lowering extracellular Ado levels, supporting a mechanistic link between ER Ca^2+^ regulation and purinergic signaling during early steatotic liver injury.

## 3. Discussion

In this study, we investigated the hepatoprotective mechanisms underlying the effects of vitamin E on HCD-induced MASLD using a transgenic zebrafish model expressing the liver-specific GRAB_Ado_ sensor, which enables the real-time visualization of hepatic eAdo dynamics in vivo [4,14]. We demonstrated that HCD feeding increased hepatic eAdo levels in a dose-dependent manner and induced steatosis, whereas vitamin E significantly reduced eAdo accumulation and ameliorated lipid deposition. VNUT inhibition, used as a positive control owing to its established ability to suppress ATP-derived eAdo release [4], resulted in similar improvements, supporting the relevance of purinergic signaling in MASLD progression. Mechanistically, although changes in inflammatory and apoptotic markers were modest, the transcriptomic analysis revealed restoration of the expression of calcium transport-related genes, particularly *atp2a1* (SERCA) and *atp1b1b*, suggesting the potential involvement of calcium homeostasis in the hepatoprotective effect of vitamin E. Furthermore, SERCA activation, mediated by the allosteric modulator CDN1163, reproduced the hepatoprotective effects of vitamin E, indicating that restoring Ca^2+^ homeostasis may function downstream of, or in parallel to, the regulation of extracellular adenosine.

The results of previous studies have shown that excessive dietary cholesterol induces hepatocellular stress, promotes DAMP release, and drives sterile inflammation, thereby contributing to MASLD and MASH progression [18,19]. Tokumaru et al. further demonstrated that VNUT-dependent ATP exocytosis is a major source of hepatic eAdo and plays a critical role in steatosis development [4]. Consistent with these findings, our results confirmed that an HCD increases hepatic eAdo in a dose-dependent manner. Importantly, we provide the first in vivo evidence that vitamin E reduces hepatic eAdo levels and alleviates steatosis to an extent comparable to that observed with VNUT inhibition. Notably, the effects of water-soluble vitamin E (tocofersolan) exhibited a plateau at 3 μM, with no further improvement observed at 6 μM. This dose–response pattern may reflect saturation of cellular uptake mechanisms or intracellular handling of vitamin E, as has been reported for vitamin E derivatives [20]. Although no overt toxicity was detected at the higher concentration in our experimental setting, it is possible that excess vitamin E does not confer additional benefit once ER Ca^2+^ homeostasis and associated stress pathways are sufficiently stabilized. Thus, the observed plateau likely reflects a limitation in bioavailability or functional efficacy rather than a simple linear dose-dependent effect. Together, these findings indicate that the hepatoprotective effects of vitamin E extend beyond its classical antioxidant and anti-inflammatory properties and involve modulation of purinergic signaling at an early stage of MASLD. To our knowledge, hepatoprotection mediated by vitamin E through regulation of extracellular adenosine has not been well characterized.

Recent evidence indicates that elevated eAdo perturbs intracellular calcium homeostasis through adenosine receptor-mediated signaling, leading to ER stress and lipid dysregulation [21,22,23]. In our study, transcriptomic profiling further revealed that vitamin E restores the expression of genes encoding calcium-handling proteins, including *atp2a1* (SERCA) and *atp1b1b*, which were downregulated with HCD-induced steatosis [24,25]. SERCA is essential for ER Ca^2+^ storage and for preventing ER stress, a key driver of lipid dysregulation and hepatocyte injury [24,25]. In the mammalian liver, ER Ca^2+^ homeostasis is predominantly maintained by SERCA2b (ATP2A2). In contrast, our RNA-seq analysis results revealed that ATP2A1 (SERCA1) is the major SERCA isoform expressed in the zebrafish liver. Moreover, *atp2a2* expression was weak, and no changes were observed in any treatment group. Moreover, *atp2a1* expression was reduced by HCD feeding and restored following vitamin E treatment, indicating that this isoform is responsive to metabolic stress and therapeutic intervention in this model. This apparent discrepancy likely reflects species-specific differences in SERCA isoform utilization in teleosts. Supporting this interpretation, public expression databases, including ZFIN (https://zfin.org/ [accessed on 2 January 2026]) and the EMBL-EBI Expression Atlas (https://www.ebi.ac.uk/gxa/home [accessed on 2 January 2026]), indicate that *atp2a1* is expressed in the zebrafish liver, whereas *atp2a2* expression is enriched in cardiac and neural tissues and remains low in the liver. Consistently, single-cell RNA-seq analyses of the zebrafish liver have revealed *atp2a1* expression in hepatocyte clusters, whereas *atp2a2* is weakly expressed or undetectable [26]. Together, these findings suggest that ATP2A1 represents the functionally dominant SERCA isoform governing ER Ca^2+^ homeostasis in zebrafish hepatocytes.

Impaired SERCA activity has been implicated in the pathogenesis of MASLD through ER Ca^2+^ depletion, activation of the unfolded protein response, and dysregulation of lipid metabolism [24,25]. Although intracellular Ca^2+^ dynamics were not directly measured in the present study, the results of previous studies indicate that HCD feeding and associated ER stress are expected to promote ER Ca^2+^ depletion and a concomitant increase in cytosolic Ca^2+^ levels [24,25]. Consistent with this framework, the recovery of SERCA-related gene expression observed in our model suggests that vitamin E may preserve ER Ca^2+^ storage capacity and mitigate ER stress, thereby interrupting the pathological cascade linking HCD-induced metabolic stress to intracellular Ca^2+^ dysregulation. Our data further suggests a bidirectional relationship between SERCA activity and eAdo signaling. Vitamin E treatment was associated with reduced eAdo levels and restoration of SERCA expression; in comparison, pharmacological activation of SERCA with the small-molecule activator CDN1163 likewise resulted in a reduction in eAdo. These findings support a model in which SERCA dysfunction and purinergic signaling form a pathological feedback loop during HCD-induced hepatic stress. Reduced SERCA activity may exacerbate ER stress and promote ATP release, leading to increased eAdo accumulation, while restoration or activation of SERCA function—either by vitamin E or by CDN1163—may stabilize ER Ca^2+^ homeostasis and attenuate stress-induced ATP/adenosine release. In this context, the ability of CDN1163 to phenocopy the protective effects of vitamin E highlights intracellular Ca^2+^ homeostasis as a critical regulatory node in eAdo-associated steatosis. Thus, vitamin E may exert hepatoprotective effects partly through modulation of the eAdo–Ca^2+^ axis, extending beyond its classical antioxidant and anti-inflammatory properties [27,28]. To date, no evidence suggests that vitamin E directly modulates purinergic receptor expression or signaling sensitivity, and this possibility was not addressed experimentally in the present study. Further studies will be required to elucidate the precise molecular mechanisms by which vitamin E modulates SERCA activity, intracellular Ca^2+^ homeostasis, and purinergic signaling.

Several limitations of this study should be acknowledged. First, intracellular Ca^2+^ dynamics were not directly assessed, and future studies employing live-cell Ca^2+^ imaging will be required to define the precise temporal relationship between SERCA activity, cytosolic Ca^2+^ levels, and eAdo accumulation. Second, the vitamin E formulation used in this study (tocofersolan) is hydrolyzed to α-tocopherol in vivo; however, its actual hepatic concentration in zebrafish larvae remains undetermined. Lastly, while zebrafish provide a powerful platform for visualizing purinergic signaling, additional validation using mammalian MASLD models will be necessary to confirm the generalizability of the eAdo–Ca^2+^ regulatory mechanism.

In conclusion, we have demonstrated that vitamin E mitigates early-stage MASLD in zebrafish by decreasing hepatic eAdo and alleviating steatosis. Transcriptomic and pharmacological evidence further suggests that the restoration of SERCA-dependent Ca^2+^ homeostasis represents a downstream or complementary mechanism of this protective effect. Although further work is required to define the precise molecular interactions, our findings reveal a previously unrecognized mechanistic link among vitamin E, eAdo signaling, and intracellular calcium regulation in MASLD pathogenesis. Combined with the utility of our hepatic GRAB_Ado_-expressing zebrafish line, this study provides a valuable platform for dissecting purinergic and Ca^2+^-dependent pathways and supports the potential of vitamin E as a therapeutic candidate targeting the earliest molecular disturbances in MASLD.

## 4. Materials and Methods

### 4.1. Zebrafish Maintenance

All zebrafish experiments were conducted in accordance with institutional and national ethical guidelines and complied with the ARRIVE guidelines. Experimental protocols were approved by the Institutional Review Board of Oita University (approval numbers: 240301, 4-34). Adult zebrafish (AB strain; ZFIN, Eugene, OR, USA) were maintained with a 14 h light/10 h dark cycle at 28–29 °C. Embryos were collected and kept in egg water at 28.5 °C until use. For all experiments, healthy larvae at 5 dpf were randomly allocated to experimental groups. A transgenic line expressing a liver-specific GRAB_Ado_ sensor, Tg (fabp10:GRAB_Ado_), was used as previously described [4]. Control larvae were fed a standard diet (Hikari Labo 130; Kyorin, 0.3 mg/larva). For MASLD induction, larvae were fed an HCD consisting of a control diet supplemented with 10–50% (*w*/*w*) cholesterol powder (FUJIFILM Wako Pure Chemical Corporation, Osaka, Japan). Larvae were fed from 5 to 9 dpf, and the food was renewed daily. For vitamin E experiments, wild-type larvae fed with 30% HCD were simultaneously exposed to water-soluble vitamin E (tocofersolan; MedChemExpress, Monmouth Junction, NJ, USA) at 0, 0.5, 1, 3, or 6 µM from 5 to 9 dpf. Egg water (with or without vitamin E) was refreshed daily after feeding. To suppress ATP-derived eAdo release, HCD-fed larvae were treated with 125 µg/mL of the VNUT inhibitor (Tokyo Chemical Industry, Tokyo, Japan) or vehicle from 5 to 9 dpf. The medium was renewed daily after feeding. For SERCA activation experiments, HCD-fed larvae were exposed to CDN1163 (0.1 or 1 µM; Sigma-Aldrich, St. Louis, MO, USA) from 5 to 8 dpf, with daily medium replacement after feeding.

### 4.2. Live Imaging and GFP Detection in GRAB_Ado_-Expressing Zebrafish

GRAB_Ado_-expressing larvae at 9 dpf were anesthetized, placed on glass-bottom dishes, and embedded in 1.5% low-melting-point agarose for live imaging. Time-lapse fluorescence images were acquired using a confocal microscope (FluoView FV3000; Olympus, Tokyo, Japan) equipped with 10× (NA 0.3) or 20× (NA 0.5) water-immersion objectives. Sequential line scanning was performed to capture fluorescence and differential interference contrast (DIC) channels. Z-series were collected with a 208 μm pinhole and 3–4 μm step sizes and then stacked using FluoView FV3000 software (Olympus). Fluorescence/ratiometric images were overlaid onto single-plane DIC images. GFP fluorescence in four anatomical regions was quantified via ROI analysis using cellSens (Olympus).

### 4.3. Histological Analysis

The larvae were fixed in 4% paraformaldehyde (4 °C, 24 h), embedded in paraffin, and processed using standard histological workflows. Consecutive 5 μm sections were H&E-stained. All images were obtained using the colored brightfield of a Keyence fluorescence microscope (Keyence, Osaka, Japan).

### 4.4. Oil Red O Staining

The zebrafish larvae were fixed overnight in 4% paraformaldehyde (4 °C), washed twice with PBS, and stained with 0.3% Oil Red O under gentle agitation (45 min). This procedure was followed by washing the sample with PBS-T and 60% isopropanol. Thereafter, the samples were placed in 50% glycerol. Lastly, the samples were placed in 3% methylcellulose and were imaged using a Leica M205 FA fluorescent stereo microscope (Leica Microsystems, Wetzlar, Germany).

### 4.5. mRNA Sequencing

The livers were dissected from 9 dpf-old zebrafish larvae. Total RNA was extracted from pooled liver samples (*n* > 150) using a PureLink™ RNA Mini Kit (Thermo Fisher Scientific, Waltham, MA, USA). Libraries for RNA-seq analysis were prepared using poly-T oligo-attached magnetic beads and sequenced using a NovaSeq X Plus (Illumina, San Diego, CA, USA). RNA sequencing reads were aligned to the zebrafish genome (GRCz11) and ENSEMBL v109 annotations with HISAT2 (version 2.2.1). Analysis of DEGs was conducted with DESeq2 (v1.28.1). KEGG (http://www.genome.jp/kegg/ [accessed on 16 June 2025]) set overrepresentation analysis was performed with ClusterProfiler (v4.8.1).

### 4.6. RT-PCR Analysis

Total RNA was extracted from pooled liver samples (*n* = 102–180) using a PureLink™ RNA Mini Kit (Thermo Fisher Scientific) and reverse-transcribed with the High-Capacity cDNA Reverse Transcription Kit (Thermo Fisher Scientific). Quantitative PCR was performed on a LightCycler 96 System (Roche Diagnostics, Rotkreuz, Switzerland) using KAPA SYBR^®^ Fast Master Mix (Kapa Biosystems, Wilmington, MA, USA) under the following conditions: 95 °C for 3 min; 45 cycles of 95 °C for 10 s, 63 °C for 30 s, and 72 °C for 10 s. Target gene expression was normalized to *gapdh* expression using the 2^−ΔΔCt^ method. Primer sequences are provided in Table 1.

### 4.7. Statistical Analysis

Results are presented as the mean ± standard error of the mean (SEM). All statistical procedures were performed using GraphPad Prism software (v9.5.0; GraphPad Software, San Diego, CA, USA). ANOVA with post hoc Bonferroni correction or Kruskal–Wallis test was used to determine statistical significance among three or more groups. Unpaired Student’s t-tests or Mann–Whitney U tests were used to compare statistical significance between two groups; *p*-values < 0.05 were considered significant.

## Figures and Tables

**Table 1:** Primers used for RT-qPCR of the zebrafish gene.

Gene	Forward Primer (5’–3’)	Reverse Primer (5’–3’)
*atf6*	CAGCACAGAGGCAGAACCTT	GTTCACTGCCGGGATTCTTTTC
*bcl2a*	GGGCCACTGGAAAACTGGAT	CCAAGCCGAGCACTTTTGTT
*caspase1*	TTCTCTGATGTCGTGCACCC	ATGTGATCCTCATGTGCGCA
*caspase3a*	TTTGATCGCAGGACAGGCAT	GCGCAACTGTCTGGTCATTG
*gapdh*	CTGGTGACCCGTGCTGCTTT	GTTTGCCGCCTTCTGCCTTA
*il1b*	ACTGTTCAGATCCGCTTGCA	TCAGGGCGATGATGACGTTC
*ire1*	GAAGGGTTGACGAAACTGCC	ATTCTGTGCGGCCAAGGTAA
*mmp9*	CTCGTTGAGAGCCTGGTGTT	CGCTTCAGATACTCATCCGCT
*nlrp3*	TCAGCTCTGAGTTCAAACCCC	CACCCATAGGATCAGTTTTGAGTG
*tnfα*	GCTTATGAGCCATGCAGTGA	TGCCCAGTCTGTCTCCTTCT
*xbp1*	GGAGTTGGAGCTGGAGAATCA	TCCAACCCCAGTCTCTGTCT
*atp2a1*	GTTGTCTCCCCTTTGAGCAG	TCCCAGATGCTCTTACCTTCTTC
*atp1b1b*	GAACTTCAGACCTAAGCCACC	CCTCACCTCACCCAGCTTAC

## Data Availability

The datasets generated in this study are available from the corresponding author upon request.
